# Pathogenicity and Bioinformatics Analysis of Two GI‐13 Infectious Bronchitis Virus Strains in China

**DOI:** 10.1155/tbed/8850463

**Published:** 2026-02-15

**Authors:** Juan Jin, Li Zhao, Yingjun Lv, Endong Bao

**Affiliations:** ^1^ College of Veterinary Medicine, Nanjing Agricultural University, No. 1 Weigang, Xuanwu District, Nanjing, 210095, Jiangsu, China, njau.edu.cn

**Keywords:** GⅠ-13, infectious bronchitis virus, pathogenicity, phylogeny, recombination

## Abstract

Despite long‐term vaccination and control efforts, infectious bronchitis virus (IBV) remains a major threat to the global poultry industry, largely due to its high prevalence and extensive genetic diversity. This study aimed to characterize two novel GI‐13 (4/91‐like) IBV field strains, CK/CH/JS/2302 and CK/CH/AH/2307, isolated from H120‐vaccinated broiler flocks in China, in order to elucidate their pathogenicity, genomic characteristics, and evolutionary relationships. Although both isolates belonged to the GI‐13 genotype but exhibited divergent pathogenic profiles and evolutionary patterns. CK/CH/JS/2302 exhibited higher virulence, severe respiratory symptoms, tracheal hemorrhage, kidney lesions, and 10% mortality, while CK/CH/AH/2307 induced only mild respiratory signs and slight renal swelling. Phylogenetic analysis revealed that CK/CH/JS/2302 displayed a recombinant genome involving GX‐YL5 and IBV/India/ck/01/23, in which the S1 gene was clustered within GI‐13 genotype, whereas other genes showed high similarity to domestic GI‐7, GI‐19, and GI‐22 genotypes. In contrast, CK/CH/AH/2307 showed high genomic similarity to the 4/91 vaccine strain without evidence of recombination but still impaired tracheal ciliary activity. Sequence and structural modeling of the S1 protein revealed that amino acid substitutions within hypervariable regions (HVRs) may affect receptor binding and antigenicity, potentially reducing cross‐protection from current vaccines. These findings demonstrate the coexistence of a virulent recombinant strain (CK/CH/JS/2302) and a low‐pathogenic variant (CK/CH/AH/2307) within the same lineage in China, underscoring the role of recombination and immune selection in IBV evolution. Overall, these findings emphasize the necessity for continuous molecular surveillance and genotype‐specific vaccine development to improve protection against emerging 4/91‐like IBV variants and reduce the economic losses caused by infectious bronchitis in poultry production.

## 1. Introduction

Infectious bronchitis virus (IBV), a representative member of the genus *Gammacoronavirus* within the family Coronaviridae, is one of the most important pathogens affecting the global poultry industry [1–4]. The virus spreads rapidly among flocks via the respiratory tract, causing high morbidity and economic losses due to growth retardation, reduced egg production and secondary infections [3, 5–9].

IBV exhibits broad tissue tropism, primarily targeting the respiratory tract but also affecting the kidneys, reproductive system, and gastrointestinal tract, thereby leading to complex and diverse pathological outcomes [10–14]. Since its first description in 1931, numerous serotypes and genotypes, such as 4/91, B1648, Aust T, QX, and TW, have been reported globally. In China, the predominant circulating genotypes include GI‐7, GI‐13, GI‐19, GI‐22, GI‐28, and GⅥ‐1 [15–23]. However, as a coronavirus, IBV undergoes frequent mutation, recombination, and insertion events, resulting in substantial antigenic variation and poor cross‐protection among different strains [24–26]. In recent years, numerous studies have revealed that the continuous evolution of IBV has led to the emergence of various recombinant strains worldwide, even among vaccinated flocks [14, 27]. The low‐virulence variant JX17 was identified as a recombinant of QX, TW, and 4/91 genotype strains [28]. Similarly, the strain ck/CH/LZJ/111113 originated from the recombination events between ck/CH/LDL/091022 and 4/91 strains [29]. The GVI‐I lineage showed an affinity for the respiratory tract of chickens, may be derived from recombination events between GI‐19 and CO8089L/CO8091L‐like viruses [30]. Additionally, a novel pathogenic IBV strain, LLN/111169, originated from multiple recombination events between the GI‐19 field strain and the Conn and 4/91 vaccine strains [31]. These genetic dynamics, coupled with complex vaccination strategies, often result in immunization failure and make disease prevention increasingly difficult [27–29, 32, 33].

In China, vaccination strategies for controlling IBV are highly heterogeneous and regionally variable, influenced by factors such as poultry type, production duration, and locally prevalent IBV serotypes. Broilers, with their short production cycles, are typically vaccinated with the live attenuated IBV vaccines, while breeder and layer chickens are routinely immunized with both live attenuated and inactivated IBV vaccines. Furthermore, the IBV vaccination programs often involved combinations of multiple IBV serotypes, for example, H120 vaccine is commonly used for priming, followed by LDT3, QX, 4/91, or TW vaccines, which can provide broader protective coverage [34]. Additionally, the IBV vaccines are frequently administered in combination with Newcastle disease virus (NDV) vaccines, further complicating vaccination programs and presenting more challenges for IBV vaccine research, particularly in ensuring their quality, stability, safety and efficiency.

With these challenges, this study aimed to characterize two IBV isolates obtained in 2023 through clinical observation, pathological assessment, phylogenetic and recombination analyses. These results provide valuable insights into the molecular evolution and epidemiological dynamics of 4/91‐like IBV variants, highlighting the necessity for continuous genomic surveillance, vaccine design, and immunization strategies to improve the control of disease in poultry populations.

## 2. Materials and Methods

### 2.1. Ethics Statement

Sample collection and animal experiments were approved by the Institutional Animal Care and Use Committee (IACUC) of Nanjing Agricultural University. The experiments were conducted in accordance with the approved guidelines.

### 2.2. Samples Collection

The samples were collected from broiler farms in Jiangsu and Anhui Provinces, where clinical disease occurred, including reduced feed intake, cough and high mortality after H120 vaccination. Tracheas and kidneys from affected chickens were collected for virus isolation.

### 2.3. Isolation of IBV

Viral isolation was performed as previously described [35–37]. Briefly, the collected samples, such as tracheas and kidneys, were individually homogenized and filtered through Sterile Filter (22 μm, Merck Millipore, Carrigtwohill, Ireland), and then inoculated in 9–10‐day‐old specific pathogen‐free (SPF) chicken embryos. The embryos were incubated for 144 h at 37°C and candled daily. The sample was considered positive for IBV if the embryos showed typical lesions, such as stunting and curling [35, 38]. The IBV‐positive samples were blind‐passaged three times in SPF embryos before titration; subsequently, two more passages were carried out to prepare the virus for animal challenge. For titration, the samples were serially diluted by 10‐fold (upto 10^−7^) in phosphate buffered saline (PBS, pH 7.2–7.4). Each dilution was inoculated into five SPF embryos, which were then observed for 144 h post‐inoculation. The 50% embryo infectious dose (EID_50_) of virus was determined with preceding steps and calculated using the Reed–Muench method [39].

### 2.4. Nucleic Acid Extraction and Reverse Transcription Polymerase Chain Reaction (RT‐PCR)

RNA was extracted from the samples using TRIzol reagent (TaKaRa, Japan) according to the manufacturer’s instructions. Briefly, 250 μL of each sample was lysed in 750 μL of TRIzol Reagent and incubated on ice for 10 min. Subsequently, 200 µL of chloroform was added, the mixture was incubated on ice for 5 min and centrifuged at 12,000 × *g* for 15 min at 4°C. The upper aqueous phase was transferred to a new tube, mixed with an equal volume of isopropanol, and incubated at −20°C for 20 min. The sample was then centrifuged at 12,000 × *g* for 10 min at 4°C. The pellet was washed with 1 mL of 75% ethanol, centrifuged at 7500 × *g* for 5 min at 4°C, and air‐dried briefly. Finally, the pellet was dissolved in 25 μL of nuclease‐free water. The extracted RNA was either used immediately for reverse transcription or stored at −80°C.

Complementary DNA (cDNA) was synthesized using the PrimeScript IV 1st strand cDNA Synthesis Mix (TaKaRa, Japan). Subsequent PCR was performed with IBV‐specific primers (designed by Premier 5; Table 1, Sq‐20‐F/R) using 2× Taq Master Mix (Vazyme, Nanjing, China). The reaction mixture consisted of 12.5 μL of 2× Taq Master Mix, 1 μL of each primer (Sq‐20‐F/R), 2 μL of cDNA, and 8.5 μL of nuclease‐free water. The thermal cycling conditions were as follows: an initial denaturation at 95°C for 3 min; followed by 35 cycles of 95°C for 15 s, 60°C for 15 s, and 72°C for 90 s; with a final extension at 72°C for 5 min, and holding at 4°C. In addition, viral suspensions were tested to exclude co‐infection with other common avian pathogens, including NDV, infectious laryngotracheitis virus (ILTV), avian leukosis virus (ALV), and fowl adenovirus (FAdV).

**Table 1:** Information of primers.

Primers	F (5′ − 3′)	R (5′ − 3′)	*T*_m_ (°C)	Position in genome^a^
Sq‐1	CAAAACGGACTTAAATACCTACAGC	CAACCACAACTGTAATTGATCTTGC	57	62–1213
Sq‐2	TGAATTGCCACAACGTATTGC	CATCCTGGATTTGAACCATATG	55	1140–2446
Sq‐3	TGTGATACTTCCAGAAAATCAACCTG	AGGTGTGACAAGTCTTTGTTGTGG	57	2403–3726
Sq‐4	CGCCGCAAATGAGCATATGTC	GCTGTAATGTAAGGCGACCTCTTCC	59	3598–4865
Sq‐5	AGTGGACATTGTTATACTCAAGCTG	ACCCATAAAGTTAAAATTAGCAAACAC	57	4774–6168
Sq‐6	TTTACATGGAATTGGCTTTATATGC	CTACCTCCTGATTTAACAGTTGC	55	5989–7187
Sq‐7	AAGTGAAAAATTCACCGCCAGTAG	ACAATAACCTGGTTTAGTAACCTCACA	57	7074–8010
Sq‐8	GCTATTGTAGAGGTAGTGTTTGTGAGGT	GAACTTTCTGAGCAACCTCTTCATC	59	7964–9374
Sq‐9	TGCATTACACACAGGAACTGACC	GACAACAGTTGTACACTTTACATCACTC	57	9309–10,646
Sq‐10	GTGTGTTGCCTATAGCTACAGTGC	CGTGTCAGCCGGATCTACTGC	55	10,586–11,904
Sq‐11	GTTGATGCTGTTGGCATACTATCAC	AATGGTACACCATCAACGAAGAC	55	11,851–13,295
Sq‐12	GTGTTCGTTGATGGTGTACC	CAAGAGGGTAAGCATCTATGG	54	13,281–14,887
Sq‐13	GAACCTGTGGCTGTTATGGAG	CTTTGAGGAACTATGCGAGTG	56	14,832–16,087
Sq‐14	CATACTTTAGTAATGCTCGTGT	ATTGTCTGCTAACATCTGAACT	54	15,969–17,385
Sq‐15	AAGAGTGCTAAACCTTGGCATGTTAT	CACAGATGTAGGCAGAGTTGTTTGAT	60	17,334–18,599
Sq‐16	AAGTTTCTCAGCTCTCCAGTCCATTG	CAGGCATATTATAACCACACGTCCAT	60	18,422–19,485
Sq‐17	AAGGAGTATGGTGCTAACAAGTCTAA	TGCGTAACAAACACTGTAAAGTCTGA	57	19,338–20,651
Sq‐18	TTGAAAACTGAACAAAAGACCG	TACAAAACCTGCCATAACTAACAT	55	20,247–21,979
Sq‐19	CAGTTTGTAGTGTCTGGTGG	TGCTGTAATGTCTCTTGGC	59	21,802–23,304
Sq‐20	ATGCAATTCAAGCAGATGCACA	TAGCCCCAGGAACTACTACCCA	60	22,893–24,325
Sq‐21	TATTTGTAGCATTTGTAGCACT	TTACTATTATTCATACCAGCGA	52	24,211–25,695
Sq‐22	AAGCAACAGTTTTTCCTCTC	CACCATCAACACACTCATCA	52	25,264–26,855
Sq‐23	ACCAAGGCAAAGGCTGATGAAA	TCCCCTAGTGGGCGTCCTAGTG	60	26,521–27,529

^a^Nucleotide positions genome corresponded to the sequence of IBV H120 genome (FJ888351.1).

### 2.5. Transmission Electron Microscopy (TEM)

TEM of IBV was performed as previously described [40]. Briefly, 20 μL of the viral suspension was pipetted onto a 150‐mesh carbon‐coated copper grid, absorbed for 3–5 min, stained with 2% phosphotungstic acid for 1–2 min, and examined with a transmission electron microscope (HT7800, Hitachi) after being dried at room temperature.

### 2.6. Pathogenicity in SPF Chickens

Pathogenicity of the isolates was evaluated using 7‐day‐old SPF chickens. 30 SPF chickens were randomly assigned to three groups (10 for each group): two infected groups (CK/CH/JS/2302 and CK/CH/AH/2307), a control group (PBS). Birds in the infected group were inoculated via the intranasal and ocular routes with 0.2 mL (10^6.0^ EID_50_) of viral suspension, whereas control birds received an equal volume of PBS. All animals were observed daily for clinical signs, morbidity, and mortality. At 14 days post‐inoculation (dpi), all surviving chickens were humanely euthanized.

### 2.7. Tracheal Ciliary Activity Assay

For the tracheal ciliary activity assay, 15 SPF chickens were used to each group. At 3, 7, and 14 dpi, five chickens per time point were humanely euthanized, and the tracheal ciliary activity of each chicken was performed as previously described [41, 42]. Tracheas were immersed in DMEM, and then cut into 2 mm rings. 10 rings per bird were randomly selected and examined by microscope. Ciliary activity was scored as follows: 0 = 100% activity, 1 = 76% – 99%, 2 = 51% – 75%, 3 = 26% – 50%, and 4 = 0%–25%. The mean score of 10 rings represented the ciliary damage index for each bird. The trachea, lung, and kidney of each chicken were collected and processed following established protocols. The tissues were fixed in 10% formalin for 24 h, followed by thorough washing with water and a series of meticulous dehydration steps. After paraffin embedding, the tissues were sectioned into slices ranging from 3 to 5 μm in thickness and then stained with hematoxylin and eosin (H&E) for observation under an optical microscope.

### 2.8. Viral Genome Sequencing

Twenty three pairs of primers were designed using SnapGene v6.1.2 (GSL Biotech, San Diego, USA) for genome sequencing via RT‐PCR (Table 1). Briefly, PCR amplification was performed by FastPfu DNA Polymerase (TransGen, China). The PCR products were purified with the Quick Gel Extraction Kit (TransGen, China) according to the protocols and subsequently cloned into the pEASY‐Blunt cloning Vector (TransGen, China). The ligated products were then transformed into Trans5α Chemically Competent Cells (TransGen, China). Transformed cells were plated onto LB agar containing ampicillin (100 μg/mL) and incubated for 14 h. Individual bacterial colonies were selected, expanded in LB medium, and submitted for sequencing. The sequencing reads were assembled and edited using SnapGene to reconstruct the viral genome sequence.

### 2.9. Phylogenetic and Recombination Analysis

Genome sequences of representative IBV genotypes (GI–GVII) were downloaded from NCBI GenBank. Multiple sequence alignment was performed using ClustalW in MEGA 11 and MegAlign, and phylogenetic trees were reconstructed. Genomic segments were analyzed including structural protein genes (S1, S2, E, M, and N) and nonstructural protein genes (1a, 1ab, 3a, 3b, 5a, and 5b). Reference sequences included: GI‐1 (H120, FJ888351.1), GI‐7 (TW2575/98, DQ646405.2), GI‐13 (4/91 vaccine, KF377577.1; GD17/04, MT701511.1; IBV/India/ck/01/23, OR824985.1), GI‐19 (QXL87, PP100176.1; GX‐YL5, HQ848267.1), GI‐22 (ck/CH/LSC/99I, KY799582.1; SAIBK, DQ288927.1), and GI‐28 (tl/CH/LDT3/03, KT852992.1).

Bootscan recombination analysis was performed using SimPlot v3.5.1, with parameters: window size 200 bp, step size 20 bp, 100 bootstrap replicates, Kimura 2‐parameter model, transition/transversion ratio = 2.0, and Neighbor‐Joining tree construction. Detected recombination signals were further confirmed using RDP v 5.74 (Recombination Detection Program, South Africa). The positions of recombination breakpoints were mapped along the IBV genome to identify affected genomic regions and potential parental lineages.

### 2.10. Structural Modeling and Comparative Analysis of the S1 Subunit

To elucidate structural variations among different IBV genotypes, the S1 protein sequences of isolates, CK/CH/JS/2302, CK/CH/AH/2307, and the reference vaccine strains, H120 (GI‐1 genotype), QXL87 (GI‐19 genotype), were subjected to homology modeling. The modeling was conducted using the SWISS‐MODEL server (https://swissmodel.expasy.org/), employing the crystal structure of the IBV spike protein (PDB ID: 6CV0) as the template. The predicted three‐dimensional (3D) structures were visualized and aligned using PyMOL v3.1.1 to compare conformational differences among the isolates and vaccine strains.

## 3. Results

### 3.1. Identification of IBV

In this study, two IBV strains were isolated from clinical samples and serially passaged five times in the allantoic fluid of chicken embryos. Both isolates were confirmed by RT‐PCR using specific primers, which showed distinct bands and were subsequently designated as CK/CH/JS/2302 and CK/CH/AH/2307 (Figure 1A). The two strains were inoculated into 9–10‐day‐old SPF chicken embryos, and induced typical lesions on embryos. The embryos inoculated with CK/CH/JS/2302 exhibited stunting and curling, whereas those infected with CK/CH/AH/2307 showed growth retardation (Figure 1B). For ultrastructural characterization, viral suspensions were examined by TEM, which revealed roughly spherical virions with diameters of approximately 80–100 nm (Figure 1C).

Figure 1Identification of IBV. (A) PCR assay for isolates. Lane 1–5, CK/CH/JS/2302, CK/CH/AH/2307, H120, allantoic fluid of chicken embryo, and water, respectively. Lane M, DNA marker. (B) Lesions in chicken embryos induced by isolates. The SPF embryos were inoculated with viral suspension for 2 and PBS for 1. CK/CH/JS/2302 caused growth retardation, curling, and hemorrhage in embryos, whereas CK/CH/AH/2307 induced growth retardation and hemorrhage without curling. (C) Morphology of the isolates observed by TEM (80,000×). 1 bar = 200 nm. “→” indicate viral particles.

**Figure:**
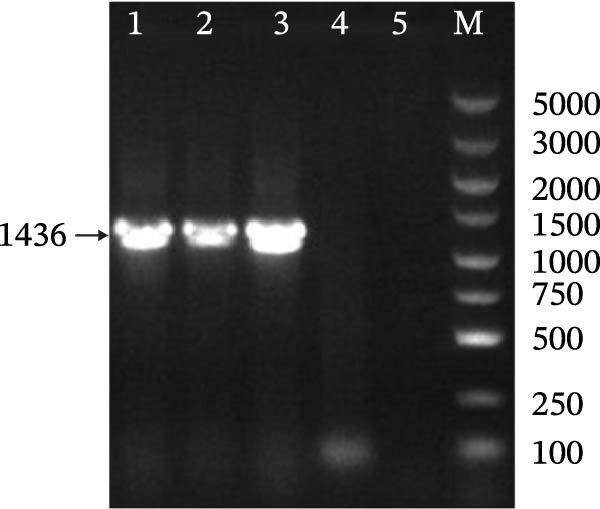
(A)

**Figure:**
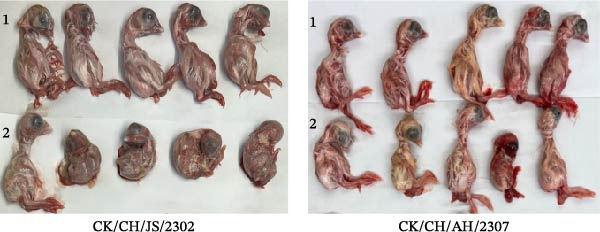
(B)

**Figure:**
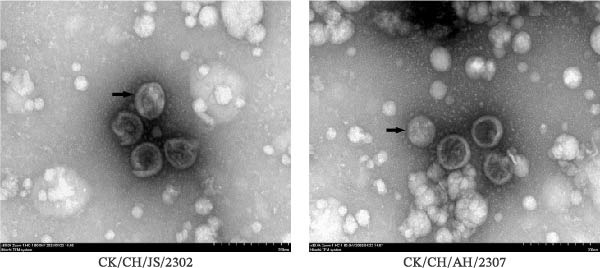
(C)

### 3.2. Pathogenicity of the Isolates in 7‐Day‐Old SPF Chickens

A 7‐day‐old SPF chickens were inoculated with the isolates and observed for 14 days. The chickens exhibited lethargy, nasal discharge and frequent head shaking, which progressed to coughing and gasping in severe cases (Figure 2).

Figure 2Pathogenicity of the isolates in 7‐day‐old SPF chickens. (A) Numbers of chickens exhibiting lethargy after infection. (B) Numbers of chickens exhibiting nasal discharge and frequent head shaking after infection. (C) Numbers of chickens exhibiting coughing and gasping after infection. (D) Survival curve of infected chicken. (E) Statistics of lesions in infected chickens, including serous exudate in tracheal lumen, petechial hemorrhages on tracheal mucosa, and swollen and pale mottled kidneys. (F) Lesions showed in infected chickens. Chicken infected with CK/CH/JS/2302 exhibited petechial hemorrhage in the tracheal mucosa, and presented with swollen, pale, and mottled kidney, whereas those infected with the CK/CH/AH/2307 only showed mildly swollen kidney and no obvious lesions in the trachea. (G) Assessment of injury to tracheal cilia induced by infection. ns, not significant;  ^∗∗^ *p* < 0.01;  ^∗∗∗^ *p* < 0.001.

**Figure:**
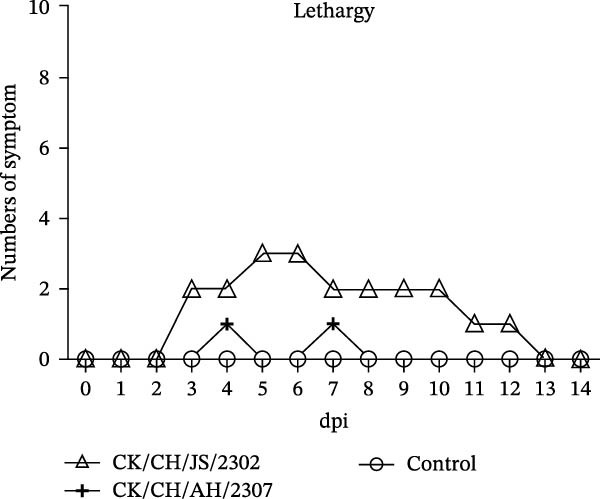
(A)

**Figure:**
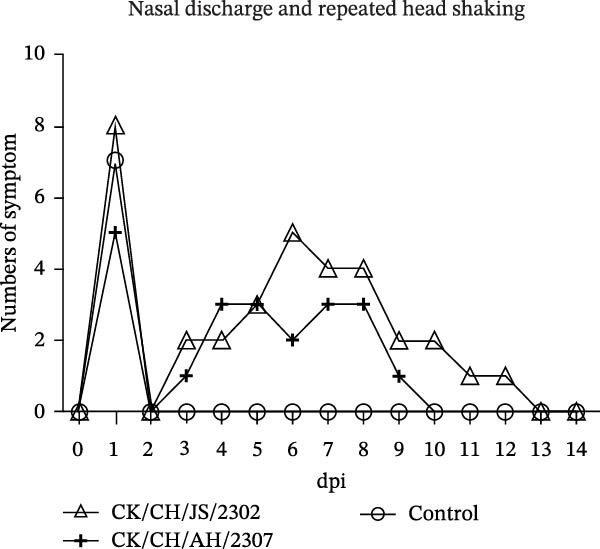
(B)

**Figure:**
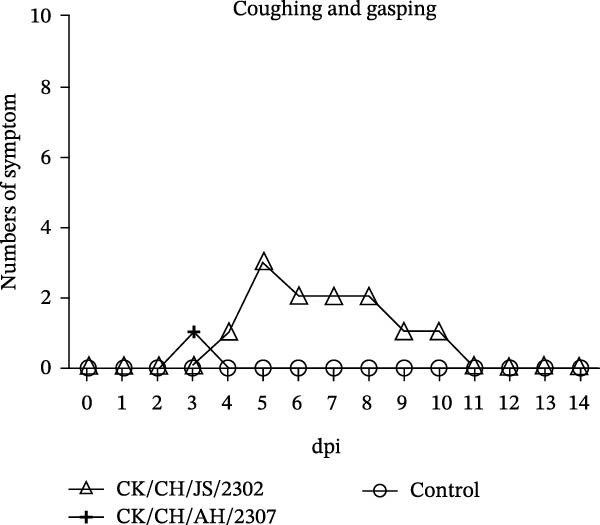
(C)

**Figure:**
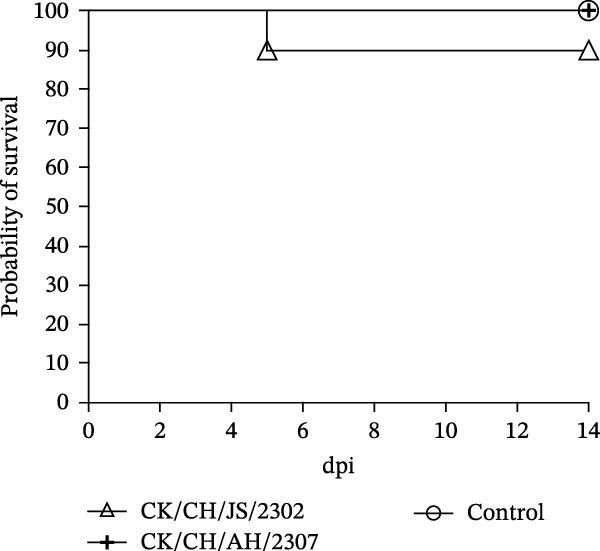
(D)

**Figure:**
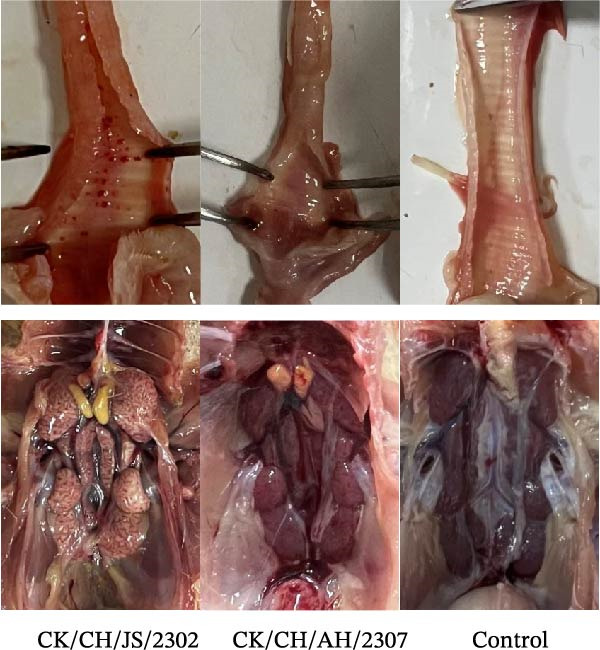
(E)

**Figure:**
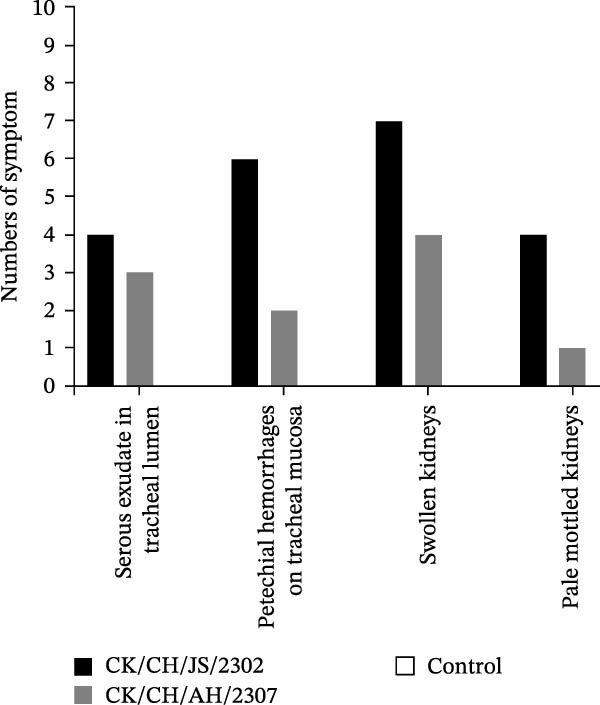
(F)

**Figure:**
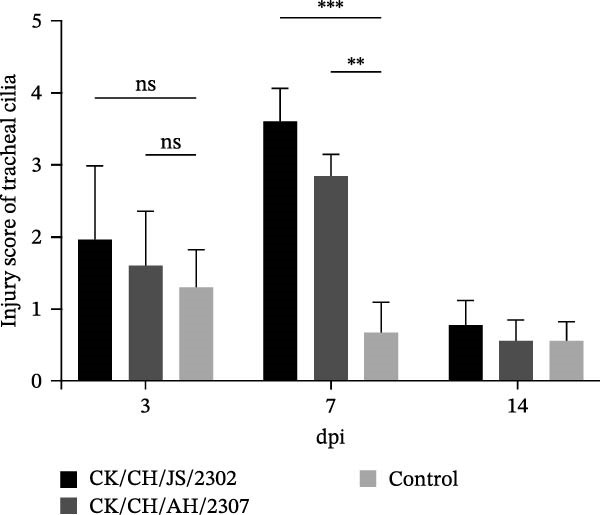
(G)

Approximately 20% of chickens in the CK/CH/JS/2302 group showed depression and lethargy at 3 dpi, increasing to 30% at 5–6 dpi, before gradually returning to normal by 13 dpi. In contrast, only one chicken in the CK/CH/AH/2307 group showed similar symptoms at 4 and 7 dpi (Figure 2A). More than 50% of chickens in this experiment exhibited frequent head shaking at 1 dpi, which resolved by 2 dpi, likely due to transient irritation caused by intranasal and ocular inoculation. Starting at 3 dpi, chickens in both infected groups developed recurrent symptoms that worsened and peaked at 6 dpi. In addition to frequent head shaking, nasal discharge was also observed. Notably, chickens in the CK/CH/JS/2302 group showed a higher incidence and longer duration of symptoms, which persisted until 13 dpi, whereas chickens in the CK/CH/AH/2307 group recovered by 10 dpi (Figure 2B). The chickens progressed to coughing and gasping when the nasal discharge increased and failed to resolve. Approximately 30% of chickens in the CK/CH/JS/2302 group exhibited these respiratory symptoms (Figure 2C), and one of them died at 5 dpi, corresponding to a mortality rate of 10% (Figure 2D). The necropsy of the dead chicken revealed petechial hemorrhages on the tracheal mucosa accompanied by serous exudation in tracheal lumen, as well as swollen, pale, and mottled kidney. In contrast, only one chicken in the CK/CH/AH/2307 group showed mild coughing at 3 dpi, with no mortality observed. Necropsy of these birds revealed only slight renal swelling (Figure 2E).

At 14 dpi, the tracheal lesions characterized by petechial hemorrhages were observed in 60% of chickens in the CK/CH/JS/2302 group, and four of which also exhibited serous exudate in tracheal lumen. Additionally, 70% of chickens in this group exhibited renal swelling, with four individuals displaying pale and mottled kidneys. In contrast, only 40% of chickens in the CK/CH/AH/2307 group showed only mild renal swelling, and 20% displayed limited petechial hemorrhages on the tracheal mucosa (Figure 2F).

Tracheal ciliary activity was assessed at 3, 7, and 14 dpi. The significant impairment of tracheal cilia occurred at 7 dpi in both CK/CH/JS/2302 and CK/CH/AH/2307 groups. However, the movement of tracheal cilia recovered to the same levels as the control group by 14 dpi (Figure 2G).

**Figure 3:**
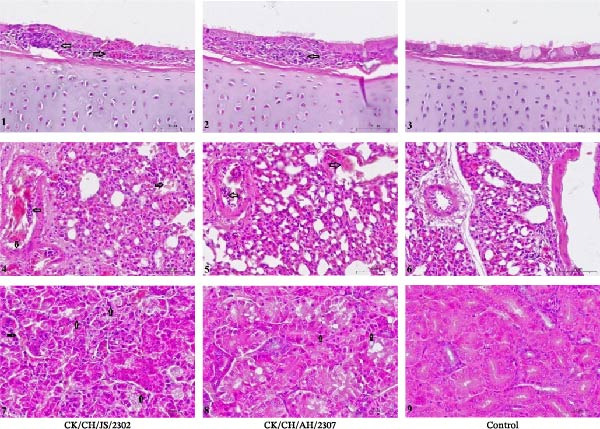
Histopathological examination of isolated viral strains in SPF chicken. Chicken, Trachea, lung and kidney; H&E staining; 1 bar = 50 μm. 1–3, Trachea: hemorrhage within the tracheal epithelium, extensive inflammatory cell infiltration, and fragmentation or sloughing of epithelial cilia; 4–6, lung: necrosis and sloughing of the mucosal epithelium in bronchi, with copious mucoserous exudate containing erythrocytes and inflammatory cells; 7–9, kidney: focal hemorrhage and widespread degeneration or necrosis of renal tubular epithelial cells. “⇦”: inflammatory cell; “⇨”: hemorrhage; “⇧”: degeneration and necrosis of renal tubular epithelial cells; “⇩”: necrosis and sloughing of the mucosal epithelium in bronchi.

Tissues collected at 7 dpi were subjected to histopathological examination (Figure 3). In the CK/CH/JS/2302 group, hemorrhage inflammatory cell infiltration was observed in the tracheal epithelia, accompanied by fragmentation and sloughing of ciliated epithelial cells. In contrast, chickens in the CK/CH/AH/2307 group exhibited only mild inflammatory cells infiltration. In the lung of chicken in the CK/CH/JS/2302 group, the bronchi showed significant necrosis and desquamation of the mucosal epithelium (Figure 3). The bronchial lumen was filled with abundant exudates containing erythrocytes and inflammatory cells. The inflammation extended centrifugally from the bronchi into the surrounding tissues, involving adjacent alveoli and leading to thickened alveolar walls and inflammatory cells exudation, consistent with bronchopneumonia. In the kidney, extensive degeneration and necrosis of renal tubular epithelial cells, along with hemorrhages were observed in the CK/CH/JS/2302 group, whereas chickens in the CK/CH/AH/2307 group displayed mild lesions in both lung and kidney (Figure 3).

### 3.3. Phylogenetic Analysis of the S1 Gene

**Figure 4:**
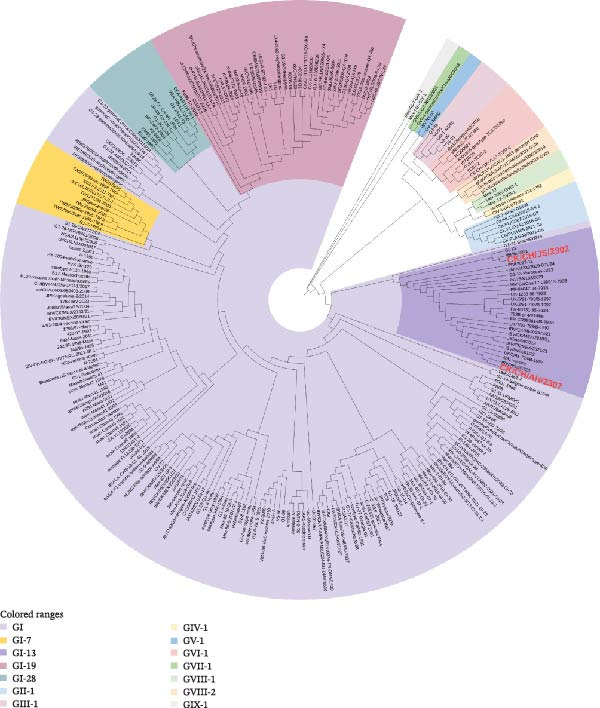
Phylogenetic tree based on the S1 gene sequences of the isolates and reference strains.

To determine the genetic classification of the isolates, the S1 gene sequences of CK/CH/JS/2302 and CK/CH/AH/2307 were aligned and analyzed using MEGA v11. Phylogenetic comparison with reference sequences downloaded from the NCBI database demonstrated that both isolates clustered within the GI‐13 genotype, confirming their classification as 4/91‐like strains (Figure 4). Notably, CK/CH/JS/2302 grouped more closely with Southeast Asian reference strains, showing high relatedness to the Vietnamese isolate IBHM‐2021, whereas CK/CH/AH/2307 exhibited high similarity with European reference strains, UK/2/91 and the 4/91 vaccine. This distinction was further supported by the sequence distance analysis (Figure S1). Specifically, CK/CH/AH/2307 exhibited S1 sequence identities of 85.5%, 96.9%, and 97.2% to IBHM‐2021, UK/2/91, and the 4/91 vaccine, respectively, whereas the corresponding values for CK/CH/JS/2302 were 95.5%, 88.9%, and 89.0%.

### 3.4. Phylogenetic Analysis of Structural and Nonstructural Protein Genes

**Figure 5:**
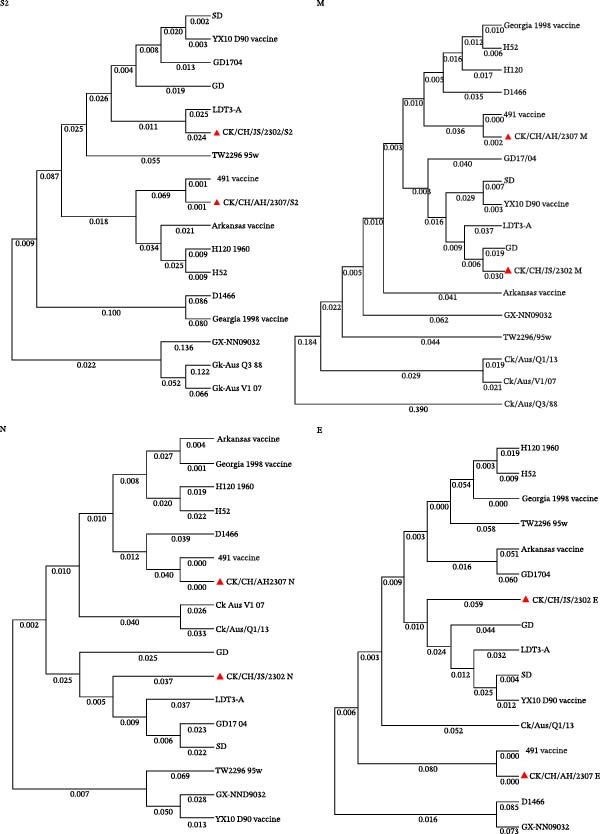
Phylogenetic trees from analysis of the structural protein genes, S2, M, N, and E. Fifteen representative IBV strains were selected, and the structural protein genes were aligned using the ClustalW in MEGA 11. Phylogenetic trees were subsequently constructed via the neighbor‐joining method to analyze their genetic evolutionary relationships.

Phylogenetic analysis based on the structural protein genes (S2, M, N, and E) revealed distinct evolutionary characteristics between the two isolates (Figure 5 and Figure S2). CK/CH/AH/2307 clustered closely with the GI‐13 (4/91 vaccine) lineage, showing sequence identities of 98.5%, 98.7%, 96.9%, and 94.6% for the S2, M, N, and E genes, respectively. In contrast, the structural protein genes of CK/CH/JS/2302 displayed more complex evolutionary relationships, with the S2 gene showing a closer relationship to the GI‐22 (LDT3‐A) strain, the M gene clustering with the GI‐7 (GD) strain, and the N and E genes exhibiting relatively close relationships to multiple domestic lineages, including GI‐7 (GD), GI‐22 (LDT3‐A), GI‐19 (SD), and GI‐13 (GD17/04). These findings suggest that CK/CH/JS/2302 has undergone multiple recombination or gene exchange events involving different genotypes.

**Figure 6:**
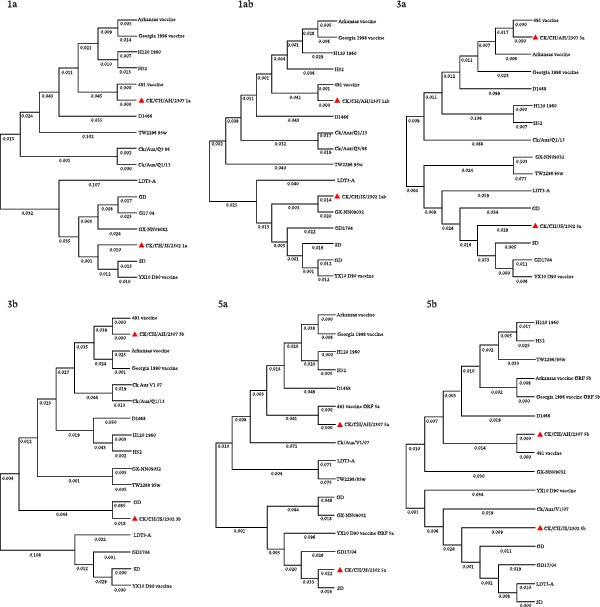
Phylogenetic trees from analysis of the nonstructural protein genes, 1a, 1ab, 3a, 3b, 5a, and 5b. Fifteen representative IBV strains were selected, and the nonstructural protein genes were aligned using the ClustalW in MEGA 11. Phylogenetic trees were subsequently constructed via the neighbor‐joining method to analyze their genetic evolutionary relationships.

Further analysis of the nonstructural protein genes (1a, 1ab, 3a, 3b, 5a, and 5b) revealed a similar trend. CK/CH/AH/2307 remained phylogenetically proximate to the GI‐13 (4/91 vaccine) lineage, whereas CK/CH/JS/2302 exhibited a complex evolutionary pattern (Figure 6 and Figure S3). Specifically, the 1a and 5a genes clustered more closely with the GI‐19 genotype (SD), the 1ab gene showed higher similarity to the GVI‐1 genotype (GX‐NN09032), the 3a gene was related to both the GI‐19 (SD) and GI‐13 (GD17/04) genotypes, and the 3b gene grouped with the GI‐7 (GD) genotype. Collectively, these results indicate that CK/CH/JS/2302 has a recombinant genomic background, reflecting extensive genetic exchanges among multiple IBV lineages.

### 3.5. Recombination Analysis

To investigate potential recombination events, Bootscan analysis was performed using SimPlot v3.5.1, with validation by RDP v5.74. CK/CH/JS/2302 exhibited high similarity to IBV/India/ck/01/23 between positions 21,000–22,250 bp (Figure 7A). Analysis by RDP v5.74 showed a potential recombination event in CK/CH/JS/2302 between GX‐YL5 (GI‐19, as the major parent) and IBV/India/ck/01/23 (GI‐13, as the minor parent), and recombination breakpoints were identified at nucleotide positions 21,145 and 22,244 (Figure 7C,D). In contrast, CK/CH/AH/2307 exhibited high sequence similarity to the 4/91 vaccine strain (KF377577.1). In particular, comparative genomic and recombination analyses revealed strong nucleotide similarity across regions 4750–10,000, 14,000–19,000, 22,000–25,750, and 26,000–27,500 (Figure 7B). Verification by RDP confirmed that no recombination events were detected between CK/CH/AH/2307 and all of the representative reference strains.

Figure 7Analysis of recombination events between isolates and representative IBV strains. (A) BootScan analysis of CK/CH/JS/2302 performed using SimPlot v3.5.1, showing a region of high nucleotide similarity with IBV/India/ck/01/23 between positions 21,000–22,250 bp. (B) BootScan analysis of CK/CH/AH/2307 by SimPlot v3.5.1, showing high nucleotide similarity with 4/91 vaccine strain but no evidence of recombination. (C) Recombination detection by RDP v5.74, indicating a potential recombination event in CK/CH/JS/2302 between GX‐YL5 (as the major parent) and IBV/India/ck/01/23 (as the minor parent). (D) Phylogenetic tree topology tests supporting the recombination signals identified by RDP v5.74.

**Figure:**
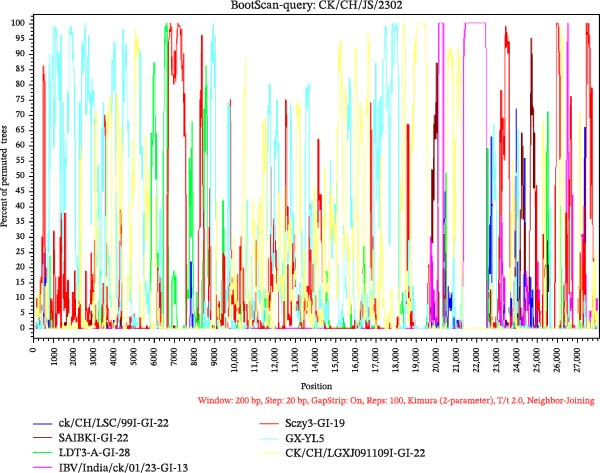
(A)

**Figure:**
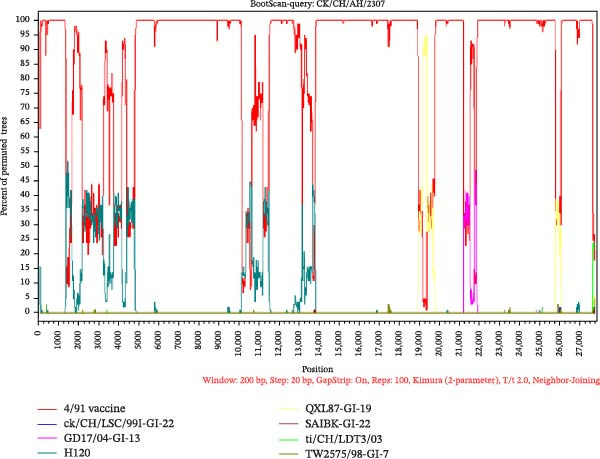
(B)

**Figure:**
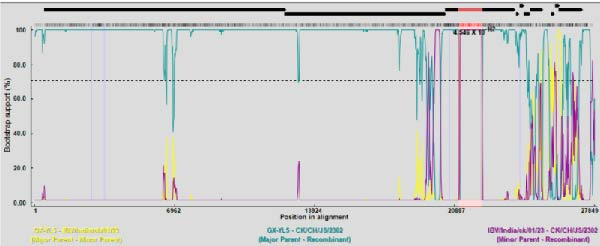
(C)

**Figure:**
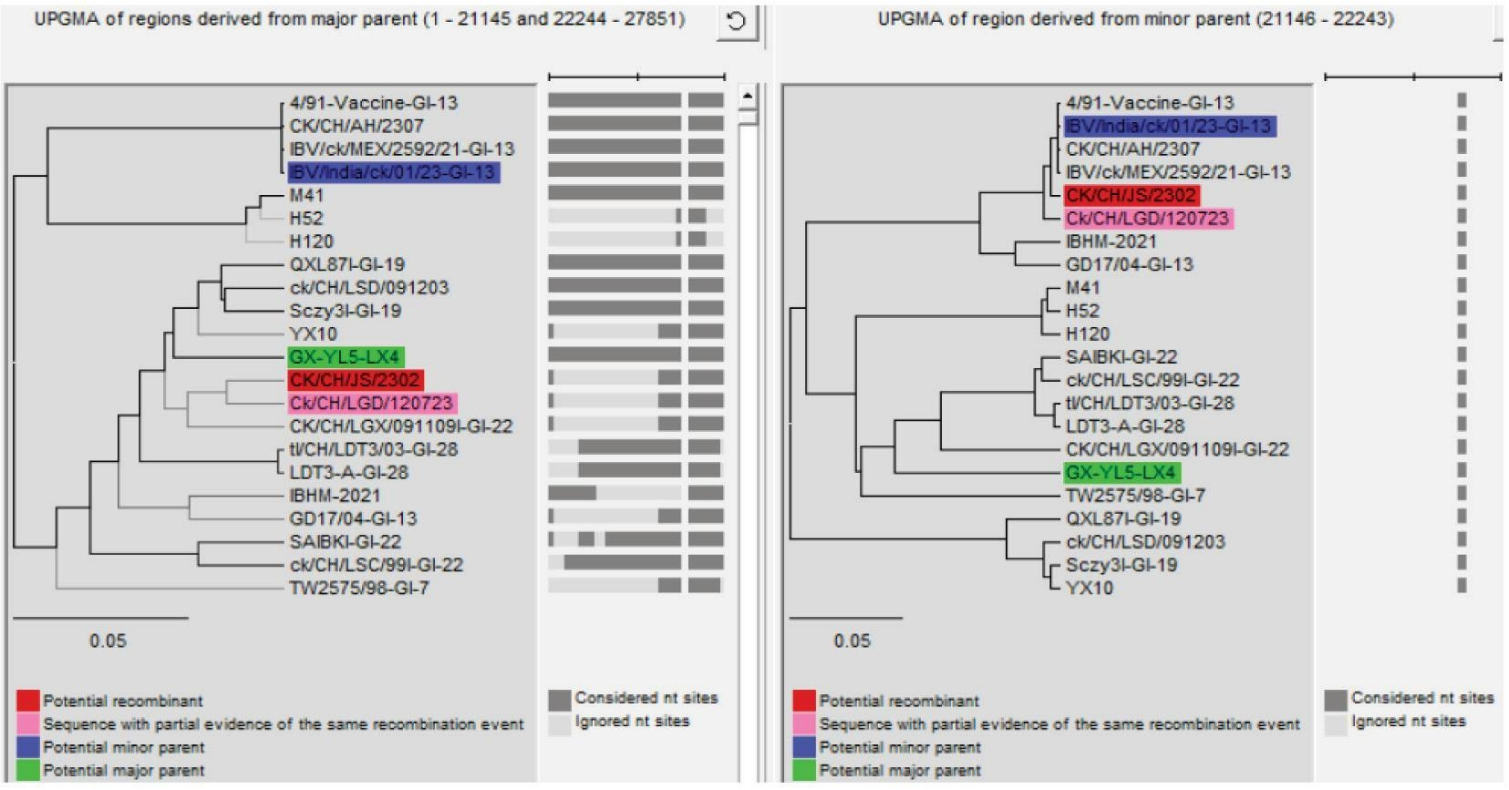
(D)

### 3.6. Amino Acid Sequence Analysis and 3D Structural Alignment

Phylogenetic analysis based on the amino acid sequences of the structural proteins (S2, M, E, and N) showed that CK/CH/AH/2307 clustered closely with the 4/91 vaccine (KF377577.1), whereas CK/CH/JS/2302 showed a more complex phylogenetic pattern, sharing genetic relationships with multiple domestic reference strains, including GI‐7 (GD), GI‐22 (LDT3‐A), GI‐19 (SD), and GI‐13 (GD17/04) (Figure 8A).

Figure 8Amino acid sequence analysis and three‐dimensional structural alignment. (A) Phylogenetic trees from analysis of the structural protein (S2, M, E, and N). (B) Multiple amino acid sequence alignment of the S1 protein. (C) Structural comparison of the S1 protein among isolates and vaccine strains. The 3D structures of the S1 protein from isolates CK/CH/JS/2302 and CK/CH/AH/2307, as well as the commonly used vaccine strains H120 and QXL87, were predicted using the SWISS‐MODEL server. Structural alignment and comparative analysis were performed to assess conformational similarities and variations among the strains, and the RMSD values were showed under the 3D structures. In the model, CK/CH/JS/2302 is shown in purple, H120 in blue, QXL87 in orange, and CK/CH/AH/2307 in green. The HVR I and HVR II are highlighted in yellow.

**Figure:**
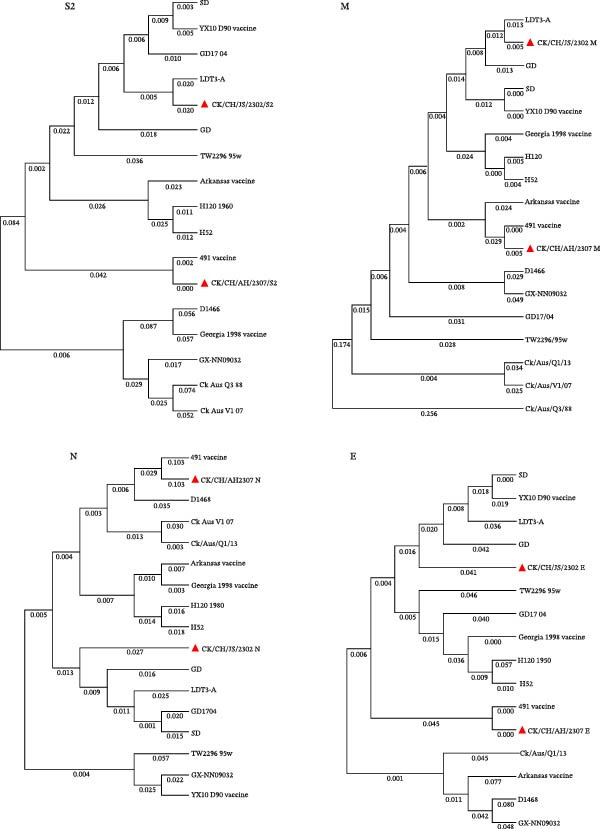
(A)

**Figure:**
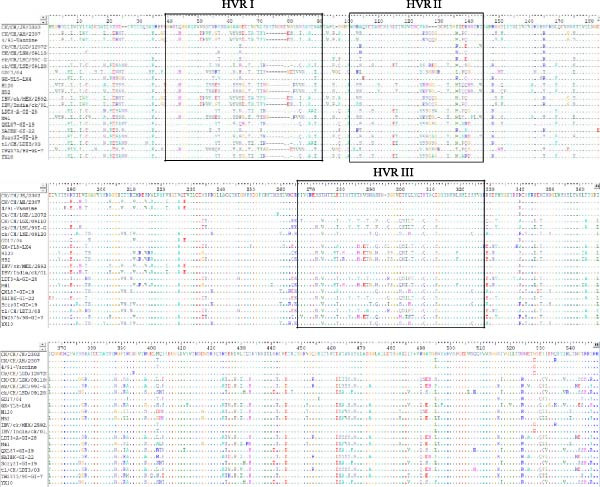
(B)

**Figure:**
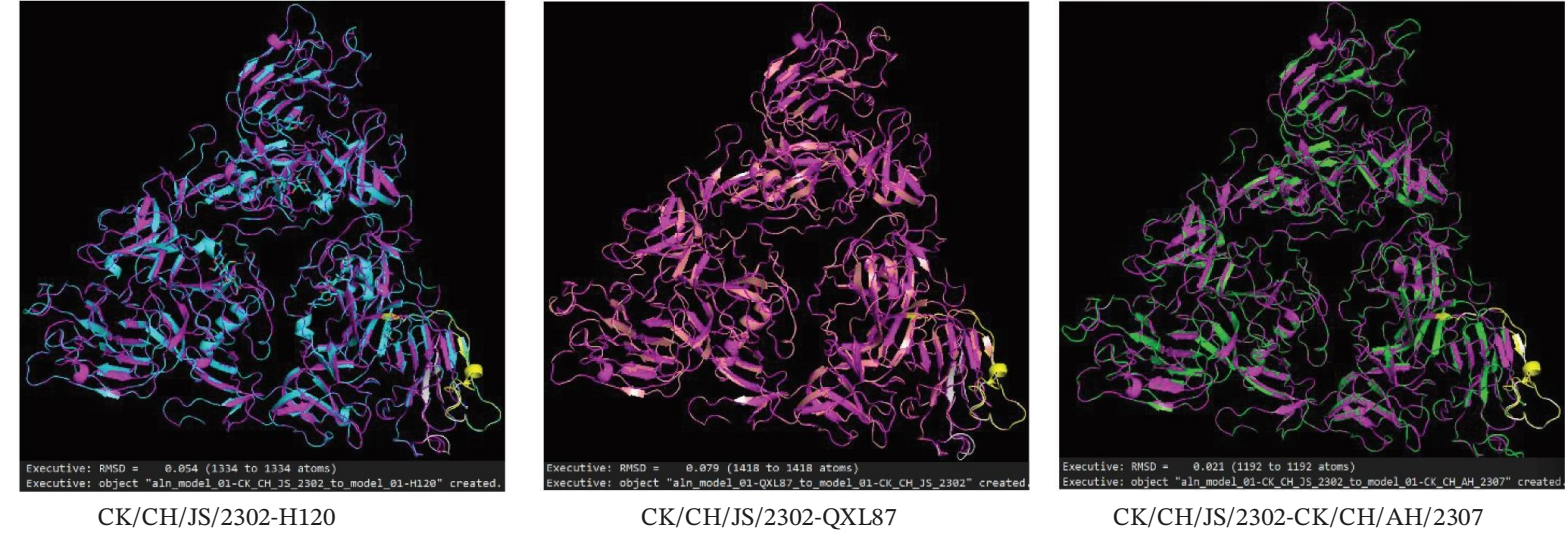
(C)

However, multiple amino acid substitutions were detected in the S1 subunit of the spike protein, mainly distributed within the hypervariable regions (HVRs) (Figure 8B). Distinct amino acid substitutions were detected at residues 51–87, 125–141, and 279–312 in CK/CH/JS/2302, while CK/CH/AH/2307 showed high sequence conservation compared with the 4/91 vaccine (Figure 8C).

To further assess the structural impact of S1 protein, the 3D structures of CK/CH/JS/2302, CK/CH/AH/2307, and the commonly used vaccine strains H120 and QXL87 were predicted using the SWISS‐MODEL server. Structural alignment and visualization were conducted with PyMOL. The calculated RMSD values between CK/CH/JS/2302 and H120, QXL87, and CK/CH/AH/2307 were 0.054, 0.079, and 0.021, respectively, indicating higher structural similarity between CK/CH/JS/2302 and CK/CH/AH/2307 (Figure 8C).

## 4. Discussion

IBV remains endemic widely in poultry flocks and causes economic losses despite decades of intensive control and vaccination. Its persistence results from high genetic diversity, frequent recombination, and broad tissue tropism, which induce immune escape and the emergence of new variants [1, 43, 44]. These evolutionary dynamics always make lower vaccine efficacy, complicate diagnosis and surveillance, and lead to difficult disease control.

Two IBV field strains, CK/CH/JS/2302 and CK/CH/AH/2307, were isolated from broiler flocks previously vaccinated with the H120 vaccine, and were characterized through pathological assessment and phylogenetic and recombination analyses. Both isolates induced respiratory disease in SPF chickens, including nasal discharge, frequent head shaking, coughing, and gasping. However, CK/CH/JS/2302 exhibited higher virulence, causing a longer duration of severe respiratory symptoms, a 10% mortality rate, and pathological lesions, including petechial hemorrhages on trachea and mottled kidney. In contrast, CK/CH/AH/2307 caused only mild respiratory symptoms and slight renal swelling. These results suggest differential virulence among 4/91‐like variants, consistent with previous reports that mutations and recombination could induce virulence variation [45–47].

Phylogenetic analysis classified both isolates within the GI‐13 (4/91‐like) genotype. However, CK/CH/JS/2302 displayed a recombinant event, with its S1 gene closely related to a Vietnamese strain (IBHM‐2021), while structural and nonstructural genes shared higher homology with diverse domestic genotypes, including GI‐7, GI‐19, and GI‐22. Recombination analysis further identified a potential recombination event in CK/CH/JS/2302 between GX‐YL5 (GI‐19, major parent) and IBV/India/ck/01/23 (GI‐13, minor parent). Such genetic exchange events are recognized as the main reason of IBV evolution, leading to altered tissue tropism and antigenicity [7, 29, 48]. Consistent with pathological assessment, the tissue damages in the trachea and kidney caused by CK/CH/JS/2302 resembled that of GX‐YL5, its putative parental strain [49]. In contrast, CK/CH/AH/2307 exhibited high genomic similarity to the 4/91 vaccine strain, with sequence identities of 97.2% for the S1 gene and 98.5%, 98.7%, 96.9%, and 94.6% for the S2, M, N, and E genes, respectively, and showed no evidence of recombination. These features are consistent with its relatively low virulence in 7‐day‐old SPF chickens. However, CK/CH/AH/2307 still induced significant impairment of tracheal ciliary activity, suggesting that it retains pathogenic potential and may have a predisposition to secondary infections, a characteristic also reported for the 4/91 vaccine strain [50, 51]. Given their close genetic relationship, CK/CH/AH/2307 may have evolved from the 4/91 vaccine strain through genetic variation following its long‐term use in China. Further pathological studies, particularly in 1‐day‐old SPF chickens, are necessary to clarify its virulence characteristics.

The S protein is recognized as the primary determinant of tissue and cellular tropism in IBV. Previous research has demonstrated that the loop structure formed by HVR within S1 protein directly mediates receptor recognition, and residues 99–159, particularly the KIP motif, are critical for renal tropism [52–55]. Comparative phylogenetic and structural analyses revealed that amino acid substitutions within HVRs I–III of CK/CH/JS/2302 induced conformational changes potentially affecting receptor binding and antigenicity. Structural modeling further showed alterations in the RBD, potentially influencing receptor affinity and antibody recognition. RMSD values demonstrated that CK/CH/JS/2302 shared the high structural similarity with CK/CH/AH/2307 but diverged from the H120 and QXL87 vaccine strains, which may suggest limited cross‐protection. Taken together, the genomic, structural, and pathogenic analyses illustrate the remarkable adaptability of circulating 4/91‐like IBV variants, shaped by the interactions between genetic evolution, immune selection, and pathogenic variation under immune pressure.

Collectively, this study provides evidence that the GI‐13 lineage in China includes both a recombinant strain (CK/CH/JS/2302) and a low‐virulent variant from vaccine strains (CK/CH/AH/2307), reflecting divergent evolutionary adaptations within the same genotype. The divergent pathogenic and genomic characteristics observed between isolates illustrate the ongoing evolution of IBV and the dynamic interactions between recombination and immune selection. Importantly, CK/CH/AH/2307 differs from the 4/91 vaccine strain and exhibits lower virulence than CK/CH/JS/2302, supporting its potential as a candidate strain for the development of next‐generation vaccines targeting endemic 4/91‐like variants that may be better adapted to field conditions in China. These findings emphasize the necessity of continuous molecular surveillance and genotype‐specific vaccine design to counteract the genetic and antigenic diversity of IBV. Effective immunization strategies based on prevalent genotypes, particularly GI‐13, are essential to enhance cross‐protection and reduce the economic impact of infectious bronchitis in poultry production.

## Funding

No funding was received for this manuscript.

## Conflicts of Interest

The authors declare no conflicts of interest.

## Supporting Information

Additional supporting information can be found online in the Supporting Information section.

## Supporting information


**Supporting Information** Sequence distances between isolates and reference strains. The representative IBV strains were selected, and the sequences were aligned using ClustalW in MegAlign to calculate sequence identity.

## Data Availability

The data that support the findings of this study are available upon request from the corresponding author. The data are not publicly available due to privacy or ethical restrictions.
