# Supplementary material for: Pathogenicity and Bioinformatics Analysis of Two GI‐13 Infectious Bronchitis Virus Strains in China
Source: Transbound Emerg Dis. 2026 Feb 15;2026:8850463. doi: 10.1155/tbed/8850463 (PMC12907510; doi:10.1155/tbed/8850463)
Supplement: Supplementary file 1 — Supporting Information Sequence distances between isolates and reference strains. The representative IBV strains were selected, and the sequences were aligned using ClustalW in MegAlign to calculate sequence identity. [file TBED-2026-8850463-s001.docx]

**Supplementary Materials**


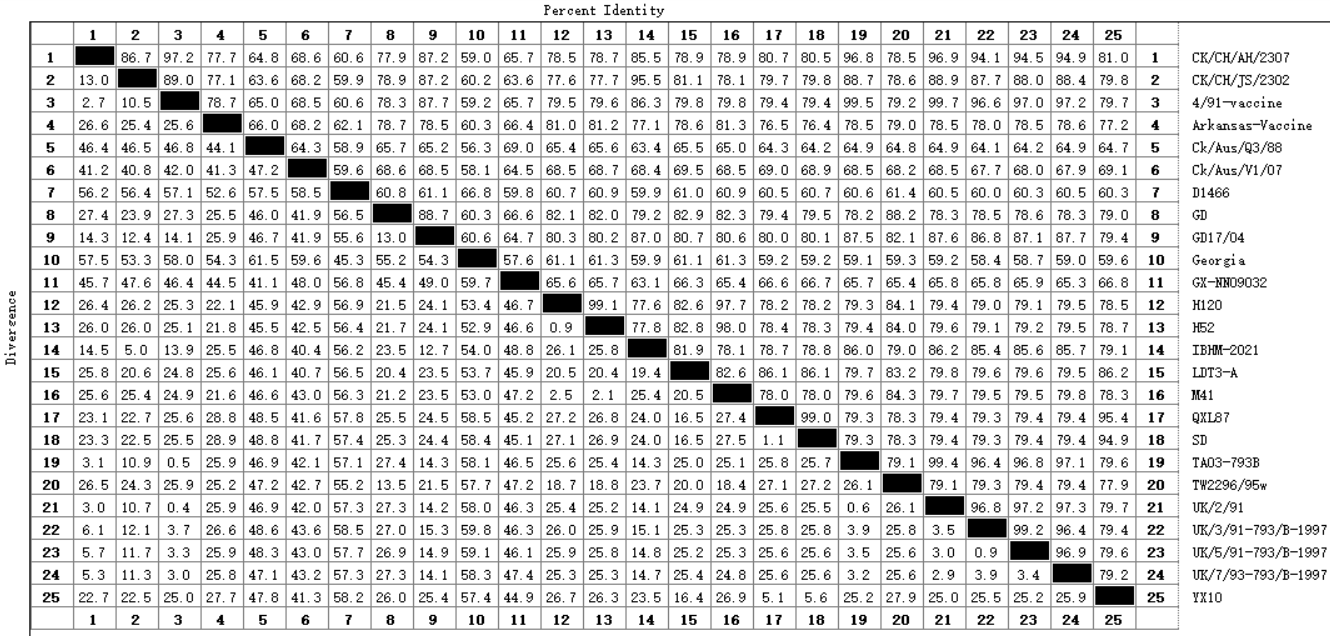


**Fig S1.** **Sequence Distances based on S1 gene between isolates and reference strains.** The representative IBV strains were selected, and the S1 protein genes were aligned using ClustalW in MegAlign to calculate sequence identity.

| S2 | 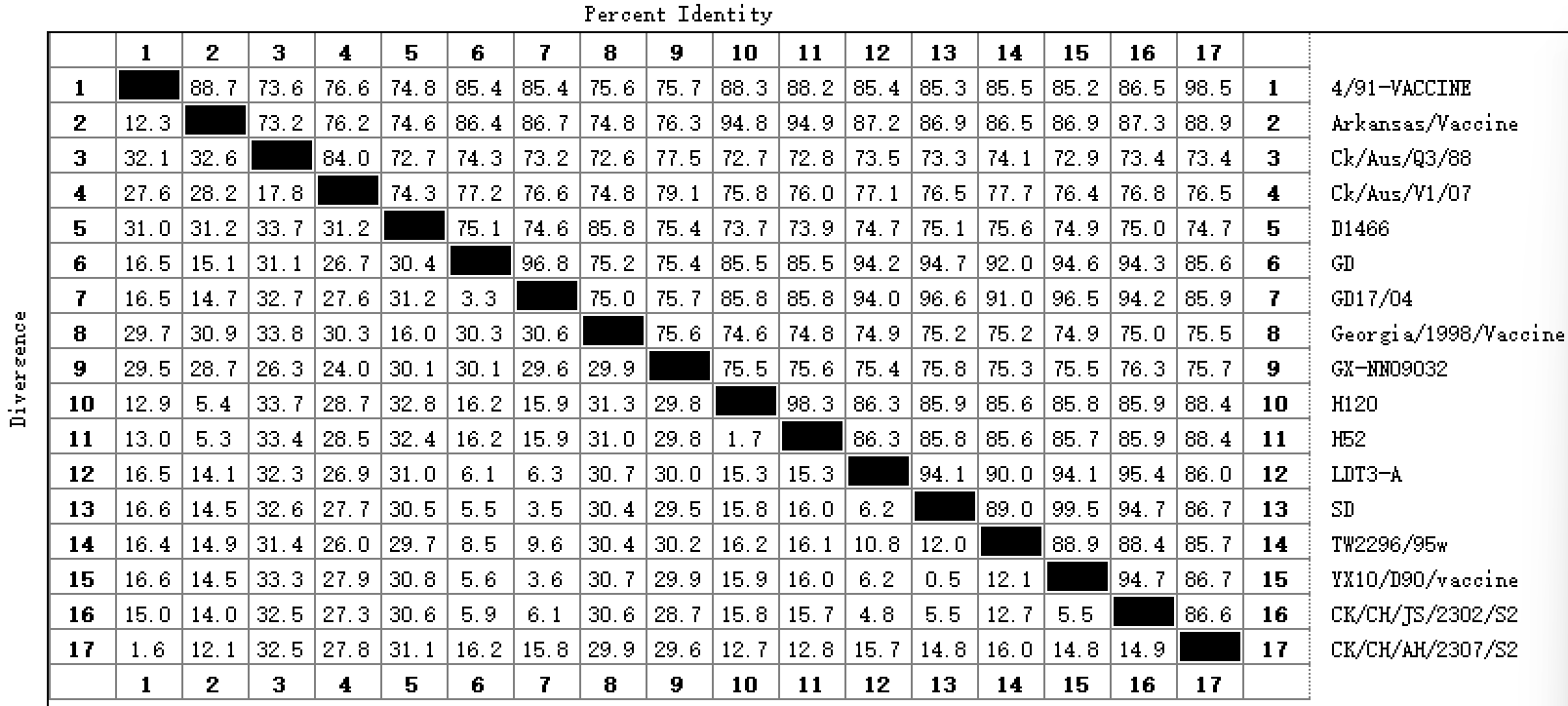 | M | 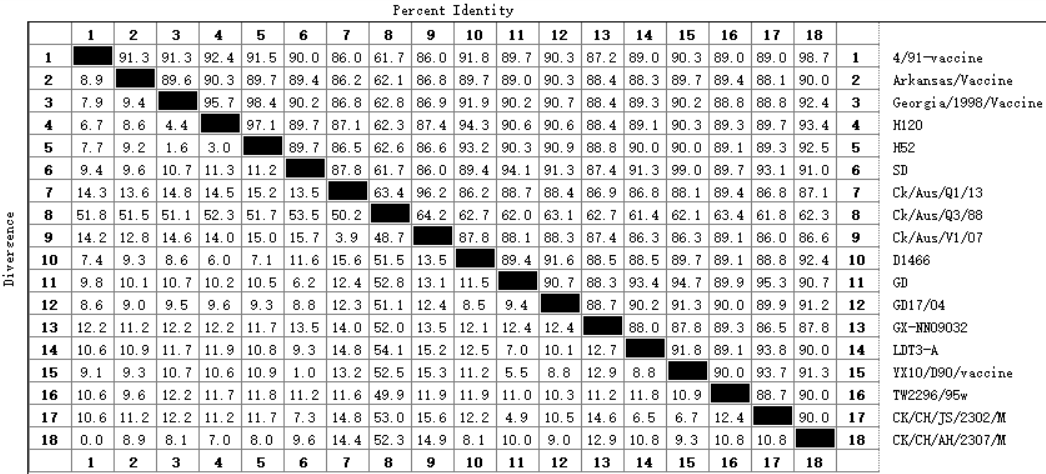 |
| --- | --- | --- | --- |
| N | 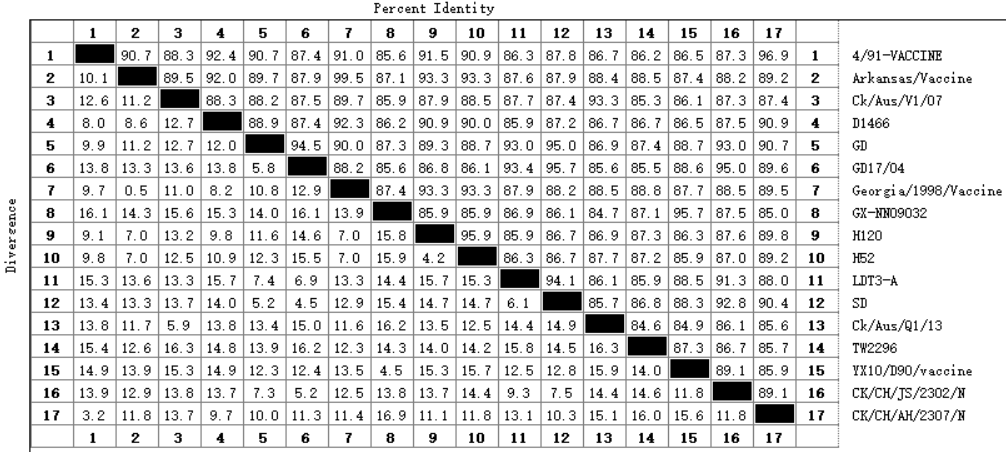 | E | 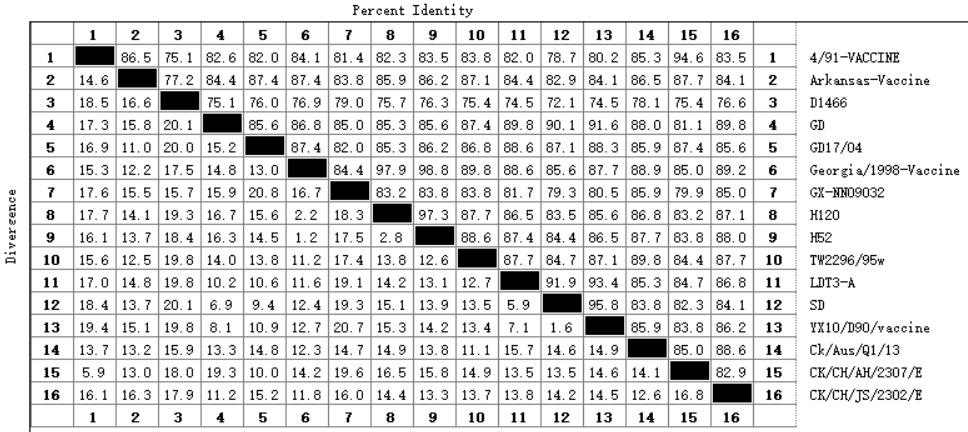 |

**Fig S2. Sequence Distances based on structural protein genes between isolates and reference strains.** The representative IBV strains were selected, and the structural protein genes were aligned using ClustalW in MegAlign to calculate sequence identity.

| 1a | 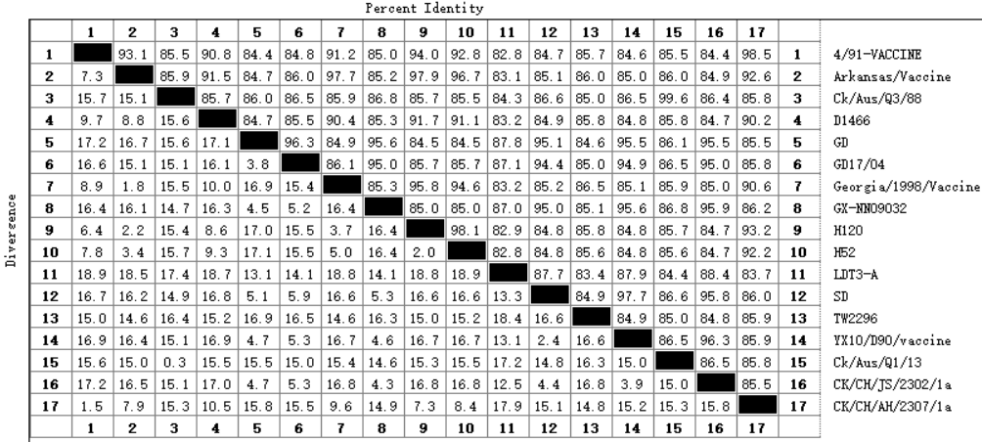 | 1ab | 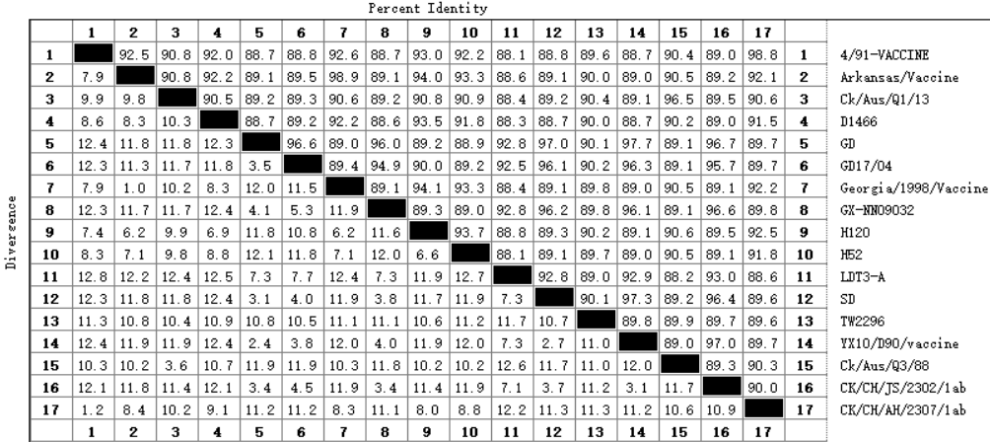 |
| --- | --- | --- | --- |
| 3a | 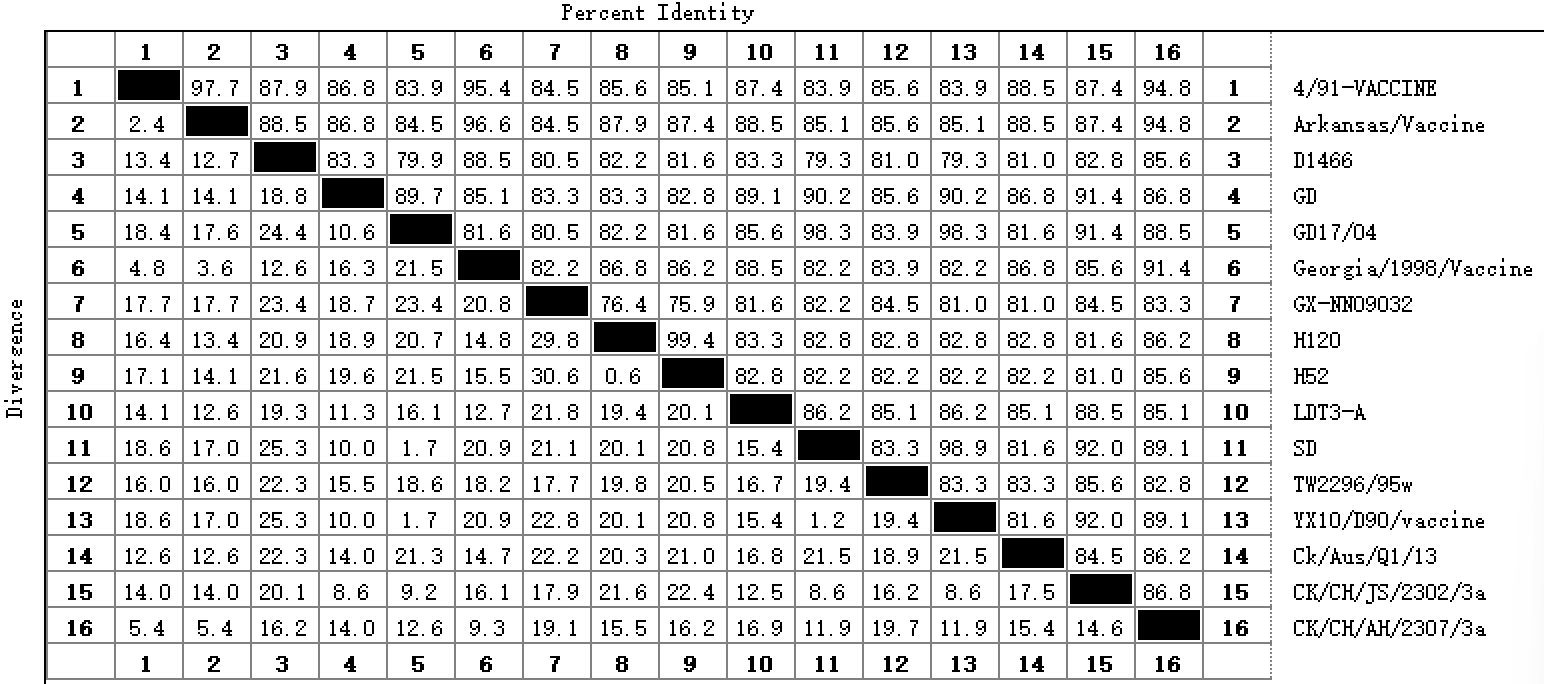 | 3b | 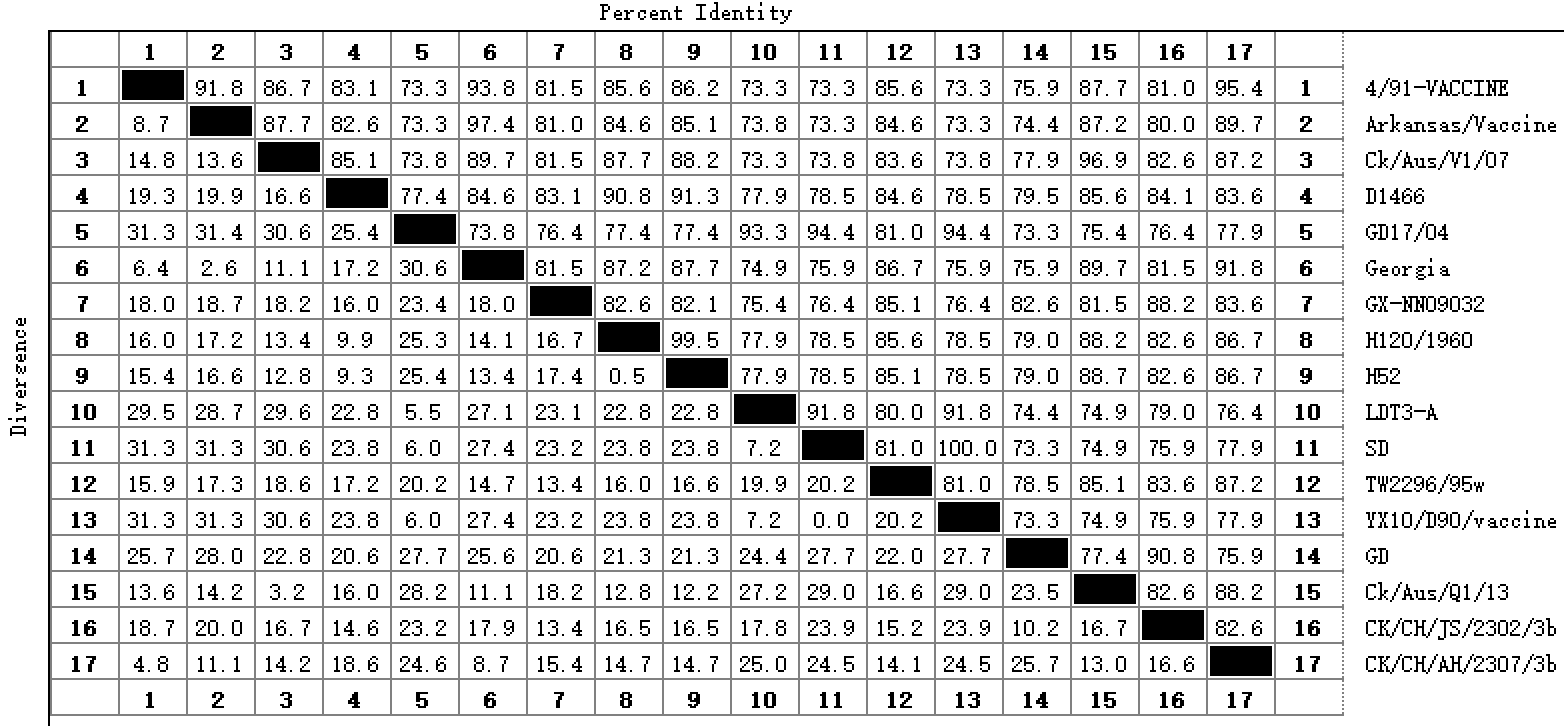 |
| 5a | 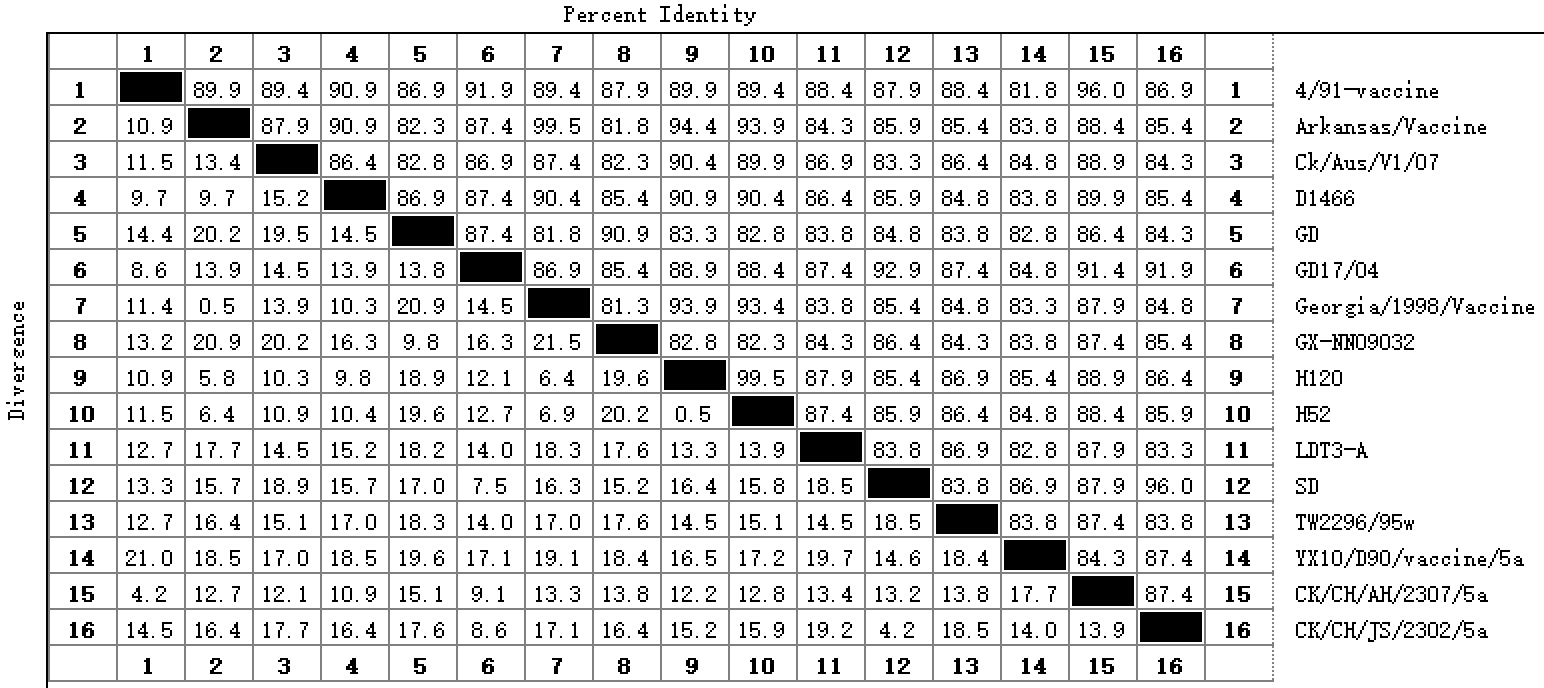 | 5b | 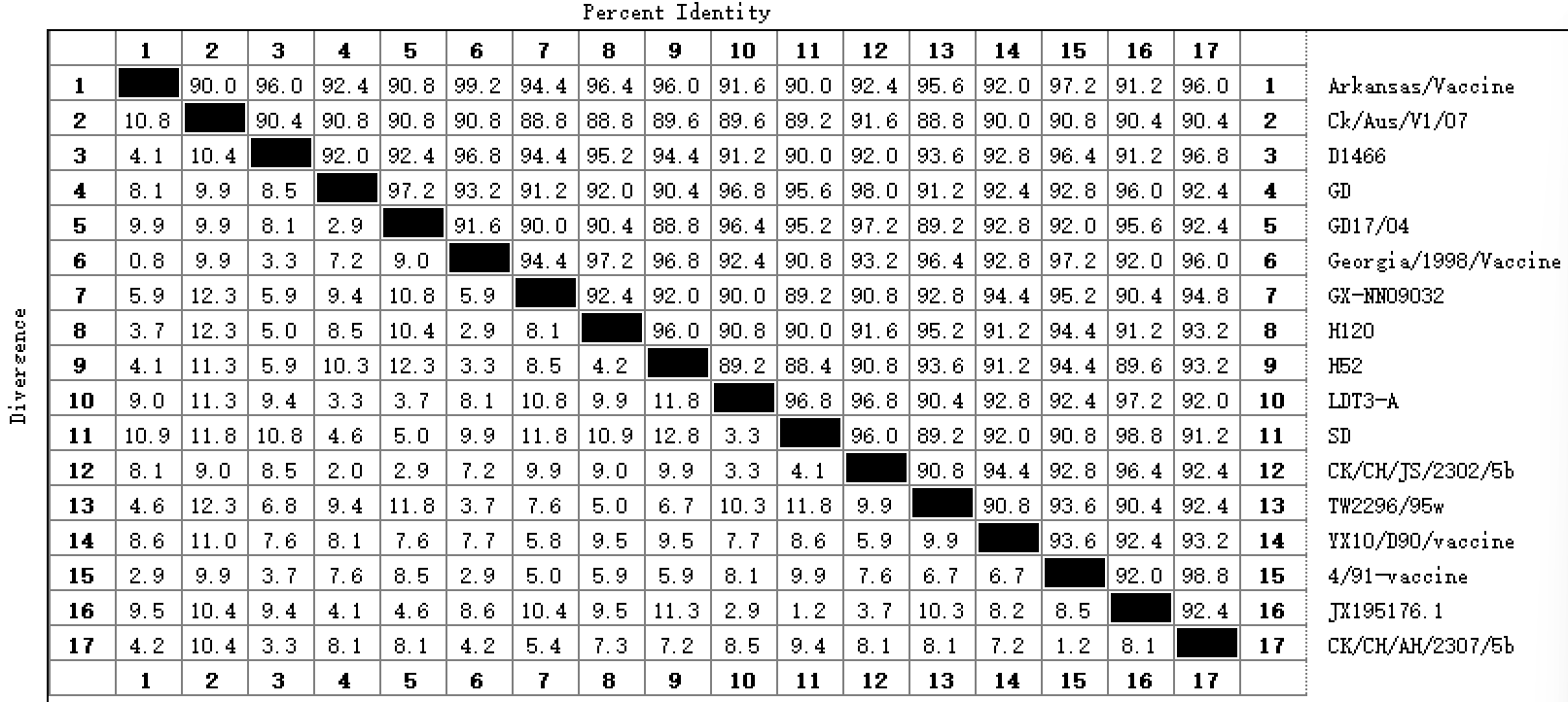 |

**Fig.S3 Sequence Distances based on nonstructural protein genes between isolates and reference strains.** The representative IBV strains were selected, and the nonstructural protein genes were aligned using ClustalW in MegAlign to calculate sequence identity.
